# Single-Cell Analysis Links C7+ Cancer-Associated Fibroblasts with Incomplete Resection in Platinum-Sensitive Relapsed Ovarian Cancer

**DOI:** 10.3390/biomedicines13123011

**Published:** 2025-12-08

**Authors:** Longxia Li, Shangbing Gao, Yilizhati Maimaiti, Lifeng Lin, Xiaoxia Xing, Wei Wu, Yulian Chen, Mei-Chun Cai, Guanglei Zhuang, Rongyu Zang

**Affiliations:** 1 Institute for Ovarian Cancer, Fudan University & Department of Gynecologic Oncology, Cancer Center, Fudan University Zhongshan Hospital, Shanghai 200032, China; llx_sync@163.com (L.L.);; 2 Shanghai Key Laboratory of Gynecologic Oncology, Ren Ji Hospital, Shanghai Jiao Tong University School of Medicine, Shanghai 200127, China

**Keywords:** relapsed ovarian cancer, secondary cytoreductive surgery, cancer-associated fibroblasts, IGF1-IGF1R

## Abstract

**Background/Objectives**: Achieving complete resection (R0) during secondary cytoreduction (SCR) is a pivotal prognostic factor for patients with platinum-sensitive relapsed ovarian cancer (PSROC). **Methods**: By integrating single-cell RNA sequencing (scRNA-seq), spatial transcriptomics, and functional assays on primary cells, we identified tumor microenvironment (TME) heterogeneity, particularly the presence of distinct subpopulations of cancer-associated fibroblasts (CAFs), as a critical determinant of surgical outcomes for SCR. **Results**: We characterized a previously unrecognized CAF-C7 subpopulation that was selectively enriched in tumors from non-R0 patients. Mechanistically, CAF-C7 promoted tumor cell migration, suppressed anoikis, and facilitated angiogenesis via the IGF1-IGF1R signaling axis, thereby contributing to multifocal recurrence and reducing the likelihood of complete resection. Importantly, the inhibition of the IGF1-IGF1R pathway effectively attenuated the pro-tumorigenic functions of CAF-C7. **Conclusions**: These findings uncover a novel cellular driver of unfavorable surgical outcomes in PSROC and suggest promising biomarkers and therapeutic targets for improving patient stratification and treatment in the context of SCR.

## 1. Introduction

High-grade serous ovarian cancer (HGSOC), the most prevalent histotype of ovarian cancer (OC), is marked by frequent recurrence and poor prognosis, making it the leading cause of death in gynecologic cancers [1,2,3]. Although cytoreductive surgery followed by platinum-based chemotherapy initially yields favorable responses, approximately 80% of patients eventually experience disease recurrence, with about 60% remaining platinum-sensitive [4,5]. Relapsed OC is categorized into platinum-sensitive (progression-free interval, PFI ≥ 6 months) and platinum-resistant (PFI < 6 months) subtypes [6,7]. Notably, platinum-sensitive relapsed ovarian cancer (PSROC), particularly at first relapse, presents a critical therapeutic window for surgical intervention.

Several landmark trials have assessed the efficacy of secondary cytoreduction (SCR) in PSROC. The DESKTOP-III trial demonstrated that patients who underwent complete resection (R0) experienced the most significant clinical benefit, with a median overall survival (OS) of 61.9 months [8]. Conversely, the GOG-0213 trial with a relatively low R0 rate reported no survival advantage with SCR [9]. We recently reported the SOC-1 study [10,11] which integrated the iMODEL [12] scoring system with PET-CT imaging to determine complete resectability, and demonstrated a 6-month OS benefit in the surgery arm. Notably, subgroup analyses across these trials consistently indicated that patients who failed to achieve R0 status had poorer outcomes compared to those treated with chemotherapy alone, highlighting the critical importance of accurate patient selection and the limitations of current preoperative assessment tools. Despite the use of integrated clinical models and advanced imaging modalities, reliable prediction of surgical outcomes remains elusive. This underscores the urgent need to identify cellular and molecular determinants that may influence the efficacy of SCR, particularly those associated with dynamic alterations in the tumor microenvironment (TME), in order to refine patient stratification and develop additional treatment strategies.

Cancer-associated fibroblasts (CAFs), as key stromal components of the TME, play multifaceted roles in tumor pathobiology by secreting cytokines, shaping the extracellular matrix (ECM), and mediating intercellular signaling [13,14,15,16]. CAFs have been shown to promote cancer cell proliferation and invasion via the secretion of pro-tumorigenic factors such as TGF-β, IL-6, and LIF [17]. Furthermore, they contribute to disease progression and therapeutic resistance through diverse mechanisms [18,19,20,21,22,23] including ECM remodeling, promotion of angiogenesis, and modulation of the immune microenvironment [19,24]. Several well-defined CAF subpopulations, including myofibroblastic CAFs (myCAFs), inflammatory CAFs (iCAFs), antigen-presenting CAFs, ECM-remodeling CAFs, IFN-responsive CAFs, and wound-healing CAFs, have been repeatedly identified in pancreatic, colorectal, breast, lung, head-and-neck, and other cancers [25,26,27,28,29]. However, the impact of CAFs on surgical outcomes in ovarian cancer has not been fully elucidated, particularly in the context of SCR for relapsed ovarian cancer.

In this study, we systematically investigated the TME of 11 patients with PSROC who underwent SCR, with a particular focus on the CAF landscape related to surgical outcomes. Using integrative multi-omics approaches and functional assays, we identified a CAF-C7 subpopulation markedly enriched in non-R0 tumors, characterized by high IGF1 expression. Mechanistic analyses revealed that CAF-C7 facilitated tumor cell migration, suppressed apoptosis, and promoted angiogenesis through activation of the IGF1-IGF1R pathway, ultimately contributing to multifocal recurrence and poor surgical resectability. These findings offer mechanistic insights into the molecular obstacles to complete cytoreduction in PSROC and provide novel biomarkers and potential therapeutic avenues to optimize preoperative decision making and improve personalized treatment outcomes.

## 2. Materials and Methods

### 2.1. Sample Collection

Fresh tumor tissues from 11 patients with platinum-sensitive relapsed ovarian cancer (PSROC) were collected as an independent cohort for single-cell RNA sequencing (scRNA-seq). Additionally, formalin-fixed, paraffin-embedded (FFPE) tissue sections (5 μm thick) from 2 PSROC patients were obtained as a separate sample set for spatial transcriptomics. Fresh tumor specimens from another 3 PSROC patients were also collected for the isolation and culture of primary cancer-associated fibroblasts (CAFs). All samples were collected at Zhongshan Hospital, Fudan University (Shanghai, China). Written informed consent was obtained from all participants. The corresponding clinical information for each sample type is provided in the Supplementary Materials: scRNA-seq samples in Table S1, spatial transcriptomics samples in Table S2, and primary fibroblast cell line samples in Table S3.

### 2.2. ScRNA-Seq

Tumor samples were enzymatically dissociated at 37 °C for 1 h using the Human Tumor Dissociation Kit (Miltenyi Biotec, Bergisch Gladbach, Germany). Following dissociation, cell viability was assessed by trypan blue (Beyotime Biotechnology, Shanghai, China) exclusion, with >80% viability confirmed. Single-cell suspensions were then used for library construction. scRNA-seq libraries were generated using the Chromium Single Cell 3ʹ Reagent Kits v3 (10x Genomics, Pleasanton, CA, USA) according to the manufacturer’s protocol. Raw sequencing data were processed using Cell Ranger (v7.1.0, 10x Genomics, Pleasanton, CA, USA) for alignment to the reference genome and gene quantification. Downstream analysis was performed in Seurat (v4.1.0, Satija Lab, New York, NY, USA), including quality control (excluding cells with >20% mitochondrial gene content or fewer than 500 or more than 6000 detected genes), data normalization, principal component analysis (PCA), and clustering based on uniform manifold approximation and projection (UMAP). Cell types were annotated by first identifying cluster-specific marker genes using the FindAllMarkers function in Seurat, followed by cross-referencing these markers with canonical gene sets reported in peer-reviewed literature to ensure accurate and biologically reliable cell type assignment [30,31]. Differential gene expression analysis within CAF subpopulations was likewise performed using the FindAllMarkers function.

The ratio of observed to expected (Ro/e) clusters [32,33] was calculated for each CAF cluster across surgical outcome groups to assess subpopulation enrichment. Expected cell numbers for each cluster and group combination were obtained using a chi-squared model, and clusters with Ro/e greater than 1 were considered enriched. For clarity, enrichment levels were categorized as +++ (Ro/e > 1), ++ (0.8–1), + (0.6–0.8), +/− (0.2–0.6), and − (0).

Intercellular communication analysis was performed using CellChat [34]. For each condition (R0 and non-R0), cells were integrated to construct condition-specific communication networks following the standard CellChat workflow. Genes expressed in more than 10% of cells within a given cluster were retained for ligand–receptor inference. Communication probability and information flow were computed using the built-in permutation-based statistical framework. Given that CellChat produces condition-level network parameters rather than per-patient replicates, the resulting communication metrics cannot be subjected to formal statistical comparison between groups. Identified ligand–receptor interactions linking fibroblast subtypes, endothelial cells, and tumor cells were visualized using ggplot2.

### 2.3. Spatial Transcriptomic

Formalin-fixed paraffin-embedded (FFPE) tissue sections (5 μm) from two patients with platinum-sensitive relapsed ovarian cancer (PSROC) were analyzed using the 10x Genomics Visium platform (10x Genomics, Pleasanton, CA, USA). Prior to spatial transcriptomic processing, adjacent H&E-stained sections were reviewed by an experienced pathologist to identify regions within the 6.5 mm × 6.5 mm Visium capture area that contained well-preserved architecture, sufficient cellularity, and representative tumor and stromal components. Only tissue areas fulfilling these criteria were selected to ensure optimal RNA capture and reliable downstream analysis. Spatial transcriptomics with the 10x Visium platform uses barcoded capture spots of approximately 55 μm in diameter. Each spot captures transcriptomic signals from multiple neighboring cells, typically 1–10 cells depending on tissue composition, rather than from individual dissociated cells.

Following standard deparaffinization and tissue permeabilization, spatially barcoded cDNA libraries were generated according to the manufacturer’s protocols. After quality control, raw sequencing data were aligned using STAR, and spot-level quantification was performed with Space Ranger (v2.1.1, 10x Genomics, Pleasanton, CA, USA) to generate gene expression matrices mapped to spatial coordinates. Spatial clustering and normalization were performed with the Spatial module in Seurat, utilizing the sctransform method. The spatial expression patterns of key genes were validated by co-registering with corresponding hematoxylin and eosin (H&E)-stained histological images.

### 2.4. Establishment of Primary Fibroblast Cell Line and Subpopulation Sorting

We established primary fibroblast cultures by isolating and expanding cells in Dulbecco’s Modified Eagle Medium (DMEM; BasalMedia, Shanghai, China) supplemented with 10% FBS (Gibco, Thermo Fisher Scientific, Waltham, MA, USA). Fresh tumor tissue was collected in DMEM on ice. On a clean bench, the tissue was washed with PBS and cut into small pieces. The pieces were mixed with collagenase (Solarbio, Beijing, China) and incubated at 37 °C for about 1 h to digest the tissue. The digested mixture was passed through a 70 µm filter (Corning, NY, USA) to remove large fragments. Cells were spun down, washed, and red blood cells were removed when needed. The final cell pellet was resuspended in complete DMEM and plated in culture dishes. After the cells were attached for 24 h, the medium was replaced with fresh medium. Fibroblast purity was confirmed after at least three passages and by immunofluorescence staining for alpha-smooth muscle actin (α-SMA; #19245, Cell Signaling Technology, Danvers, MA, USA) and fibroblast activation protein (FAP; ab218164, Abcam, Cambridge, UK). For subpopulation isolation, single-cell suspensions obtained after at least three passages were labeled with an APC-conjugated anti-human CD34 antibody (S20016E, BioLegend, San Diego, CA, USA) for 30 min at 4 °C in the dark, followed by fluorescence-activated cell sorting (FACS) using a BD FACSAria III (BD Biosciences, Franklin Lakes, NJ, USA) flow cytometer. Aliquots (1 × 10^4^ cells) were prepared by cytocentrifugation for morphological analysis, which involved 4% paraformaldehyde (Beyotime) fixation, DAPI (Beyotime) counterstaining, and fluorescence microscopy (Olympus, Tokyo, Japan) imaging.

For conditioned medium (CM) preparation, sorted CAF-C7 (CD34^+^) cells were seeded and cultured until they reached approximately 85% confluence. Cells were then washed twice with PBS and incubated in FBS-free DMEM for 48 h. The supernatant was collected, centrifuged at 1500 rpm for 5 min to remove debris, and passed through a 0.22 μm filter to obtain CM. The corresponding control medium consisted of FBS-free DMEM incubated under identical conditions but without exposure to CAFs. All CM samples were used fresh or stored at −80 °C until analysis.

### 2.5. Cell Lines and Reagents

The human ovarian cancer cell lines SKOV3 and HEK293T cells were obtained from the American Type Culture Collection (ATCC, Manassas, VA, USA). The human ovarian cancer cell line COV318 was obtained from Sigma-Aldrich (St. Louis, MO, USA). COV318 and SKOV3 were selected based on their IGF1R expression levels. Although SKOV3 is not derived from HGSOC, it is widely used and well characterized in ovarian cancer research, providing robust functional reproducibility and complementing the HGSOC-relevant COV318 model. All cells were maintained in DMEM containing 10% FBS and 1% penicillin-streptomycin (Gibco, Thermo Fisher Scientific, Waltham, MA, USA) under standard culture conditions (37 °C, 5% CO_2_). Routine testing confirmed the absence of mycoplasma in all cell lines. IGF-I/IGF-1 Protein, Human (70a.a, HY-P7018) was purchased from MedChemExpress (Monmouth Junction, NJ, USA).

### 2.6. Knockout Experiments

The sgRNA sequences targeting the IGF1R gene were designed and cloned into the pLentiCRISPRv2 vector. Lentiviral particles were generated by transfecting HEK293T cells with the constructed plasmids using Lipofectamine 3000 (Invitrogen, Thermo Fisher Scientific, Waltham, MA, USA). The resulting lentivirus was used to infect the human ovarian cancer cell lines COV318 and SKOV3 for 24 h. Following infection, cells were subjected to puromycin selection (2 μg/mL; Beyotime) until all non-transduced control cells were eliminated, ensuring stable integration of the CRISPR constructs. Knockout efficiency in both COV318 and SKOV3 cells was assessed at the protein level by Western blot analysis of IGF1R expression. Only cell populations demonstrating clear loss of IGF1R protein were used for subsequent functional assays.

### 2.7. Western Blot

Cell lysates were prepared using RIPA buffer (Beyotime) supplemented with protease and phosphatase inhibitors (Beyotime). Protein concentrations were determined using the BCA protein assay kit (Epizyme Biotechnology, Shanghai, China). Equal amounts of protein (10 μg) were separated by 10% SDS-PAGE and transferred to PVDF membranes (Millipore, Burlington, MA, USA). Membranes were blocked and subsequently incubated overnight at 4 °C with primary antibodies against IGF1R (#9750, Cell Signaling Technology). Afterward, the membranes were incubated with HRP-conjugated secondary antibodies (A0201, Beyotime) for 1 h at room temperature. Protein bands were visualized using ECL chemiluminescence reagent (Yeasen Biotechnology, Shanghai, China) on a TIANGEN gel documentation system (TIANGEN, Beijing, China). For quantification, original 8-bit TIFF files were imported into ImageJ (NIH, Bethesda, MD, USA). Background subtraction was performed using a rolling ball radius of 50 pixels, and rectangular regions of interest (ROIs) were drawn around each band. Integrated density values were extracted using the Gel Analysis module. IGF1R band intensities were normalized to GAPDH in the same lane, and the resulting ratios were further scaled to the mean IGF1R/GAPDH value of the NC group. Quantification was performed using at least three independent experiments. Statistical analysis and graphing were completed in GraphPad Prism (version 9.0, GraphPad Software, San Diego, CA, USA).

### 2.8. Anoikis Assay

IGF1R-NC and IGF1R-KO ovarian cancer cells were plated in low-attachment 24-well plates (FULA243, Beyotime) at a density of 1.25 × 10^6^ cells/well. IGF-I (100 ng/mL) or PBS was added at the time of plating and maintained throughout the 48 h incubation period.

Following treatment, suspended cells were collected and dissociated into single-cell suspensions using 0.25% trypsin. After digestion, cell numbers were assessed using Trypan Blue staining to preliminarily confirm that no differential cell death occurred across groups due to trypsinization, and equal numbers of viable cells were used for downstream analysis. Cells were then washed with PBS, stained with an Annexin V-FITC/PI apoptosis detection kit (BD Biosciences), and subsequently analyzed by flow cytometry. Three biological replicates (*n* = 3) were performed per condition. The data were analyzed using FlowJ (version 10.8.1, BD Life Sciences, Ashland, OR, USA), and statistical analyses were performed with GraphPad Prism.

### 2.9. Wound Healing Assay

Confluent monolayers of ovarian cancer cells were established in 6-well plates at a density of 5 × 10^5^ cells/well. Linear wounds were created using 200 μL sterile pipette tips, followed by treatment with either 100 ng/mL IGF-I or CAF-C7 conditioned medium (CM). Wound closure was monitored at 0, 24, and 48 h post-wounding, and images were captured under a light microscope at each time point. Scratch assay images were analyzed using ImageJ. For each image, the raw file was opened and converted to 8-bit, followed by smoothing and edge detection. A Gaussian blur (sigma = 6, adjusted as needed based on image quality) was applied to reduce noise. Automatic thresholding (Default, dark background) was then used to generate a binary mask. After converting to a mask, the wound area was selected using the wand tool at two representative points along the scratch. Regions outside the wound were cleared, and the image was inverted to highlight the wound area. The resulting region of interest (ROI) was added to the ROI Manager, and the wound area was quantified using the “Measure” function. All images were processed using identical settings to ensure consistency across experimental conditions. Each experiment was performed in triplicate, and statistical comparisons were conducted using two-tailed *t*-tests in GraphPad Prism.

### 2.10. Migration Assay

Cells (5 × 10^4^) were seeded in the upper chamber of 8 μm pore-size Trans-well inserts (Corning), with complete medium containing 15% FBS in the lower chamber as chemoattractant. After 14 h incubation, non-migrated cells were removed from the upper membrane surface. Migrated cells on the lower surface were fixed with 4% paraformaldehyde, stained with 0.1% crystal violet (Beyotime). For quantification, migrated cells were counted manually in three randomly selected fields per insert at 100× magnification, and the average number of cells per field was used for analysis. All experiments were performed in three independent biological replicates.

### 2.11. Tube Formation Assay

Matrigel matrix (100 μL/well, Corning) was polymerized in 96-well plates at 37 °C for 30 min. HUVECs (5 × 10^3^/well) were cultured with CAF-C7 conditioned medium for 6 h. Tube-like structures were imaged under an inverted microscope (40× magnification) and analyzed using the Angiogenesis Analyzer plugin for ImageJ to quantify total tube length and branching points.

### 2.12. Statistical Analysis

All experimental data represent at least three independent biological replicates and are presented as mean ± SD. Statistical comparisons between groups were performed using unpaired two-tailed Student’s *t*-tests in GraphPad Prism (version 9.0, GraphPad Software, San Diego, CA, USA). Significance thresholds were defined as * *p* < 0.05, ** *p* < 0.01, and *** *p* < 0.001. All bioinformatics analyses were conducted using R (version 4.4.1, R Foundation, Vienna, Austria) and RStudio (version 2023.06.0, Posit Software, Boston, MA, USA).

## 3. Results

### 3.1. Tumor Microenvironment Profiling and Fibroblast Subtype Analysis in PSROC

**Figure 1:**
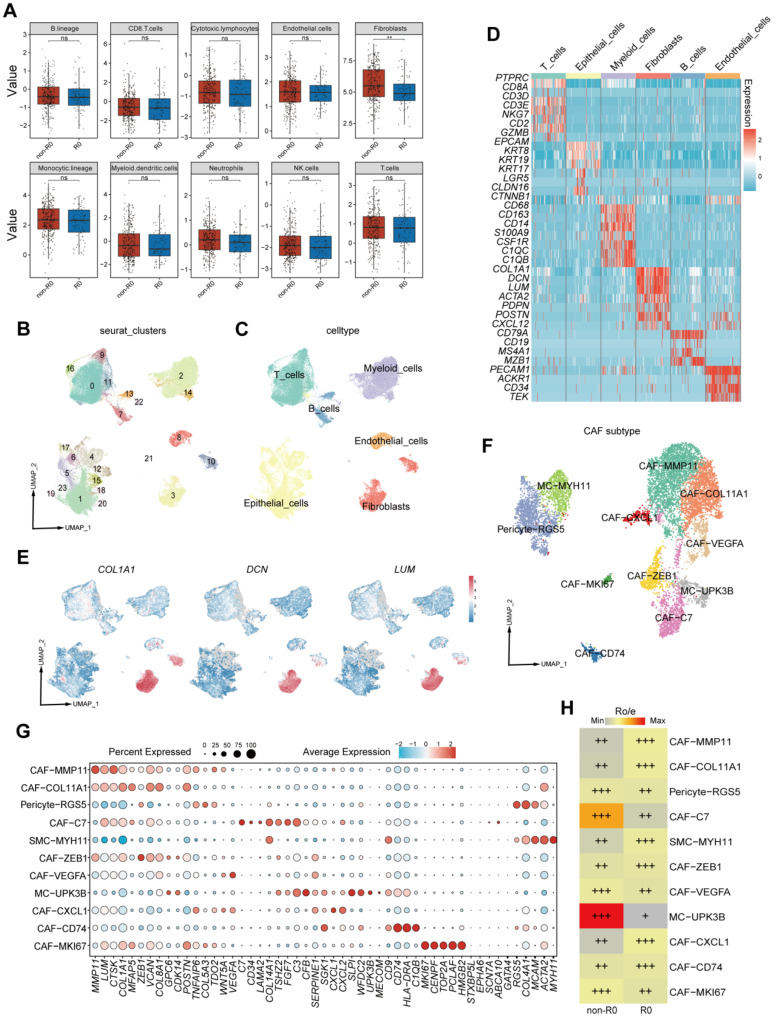
Charting the tumor microenvironment landscape of PSROC by scRNA-seq. (**A**) Quantification of absolute infiltration levels of eight immune cell types and two stromal cell types (fibroblasts and endothelial cells) in the tumor microenvironment (TME) based on ovarian cancer tissue samples from the TCGA database. ** *p* < 0.01. ns—not significant. (**B**) Uniform Manifold Approximation and Projection (UMAP) visualization showing 24 cell clusters identified through integrated analysis of samples from 11 PSROC patients. (**C**) 24 identified discrete cell clusters were annotated into six major cell lineages according to canonical marker gene expression. (**D**) Heatmap displaying the expression patterns of marker genes across different various cell types. Red indicates high expression, while blue indicates low expression. (**E**) Feature plots illustrating the expression distribution of fibroblast marker genes (*COL1A1*, *DCN*, and *LUM*) across cell clusters. The color gradient represents gene expression levels, with red indicating high expression and blue indicating low expression. (**F**) UMAP visualization of fibroblast subpopulations identified in relapsed ovarian cancer samples, with each color representing a distinct fibroblast subcluster. (**G**) Dot plot showing the expression levels of signature genes in each fibroblast subpopulation. Dot size represents the expression proportion, and color indicates the mean normalized expression level. (**H**). Ratio of observed to expected (Ro/e) analysis revealing differential distribution of fibroblast subpopulations between the non-R0 and R0.

To investigate tumor microenvironment (TME) features associated with surgical outcomes in PSROC, we employed an integrative multi-omics framework. Initial analysis of The Cancer Genome Atlas (TCGA) ovarian cancer cohort (*n* = 374; R0: 75 cases; non-R0: 299 cases) was conducted using the Microenvironment Cell Populations-counter (MCP-counter) algorithm [35] to quantify absolute infiltration levels of eight immune cell types (CD8^+^ T cells, CD4^+^ T cells, B cells, NK cells, macrophages, dendritic cells, mast cells, and neutrophils) and two stromal cell types (fibroblasts and endothelial cells). Among all evaluated populations, fibroblast infiltration was significantly elevated in the non-R0 group, while no significant differences were observed in other cell types (Figure 1A). These results implicate fibroblasts as key stromal components that may potentially impede complete cytoreduction in relapsed ovarian cancer.

To further delineate fibroblast heterogeneity, we performed single-cell RNA sequencing (scRNA-seq) on tumor tissues from 11 PSROC patients (R0: *n* = 8; non-R0: *n* = 3, Supplementary Materials, Table S1). After stringent quality control and dimensionality reduction using Uniform Manifold Approximation and Projection (UMAP), 24 discrete cell clusters were identified. Cluster-specific marker genes were first identified using the FindAllMarkers function in Se urat. These markers were then cross-referenced with canonical marker sets reported in the literature [30,31], and clusters were subsequently annotated into six major cell lineages: epithelial cells (*EPCAM*, *KRT8*, *KRT19*), T cells (*CD3D*, *CD3E*, *CD8A*), myeloid cells (*CD68*, *CD163*, *CD14*), B cells (*CD79A*, *MS4A1*, *MZB1*), endothelial cells (*PECAM1*, *ACKR1*, *CD34*) and fibroblasts (*COL1A1*, *DCN*, *LUM*) (Figure 1B–E, Supplementary Materials, Figure S1).

Unsupervised sub-clustering of fibroblasts revealed 11 transcriptionally distinct subpopulations (Figure 1F), annotated according to differentially expressed genes (Figure 1G). Their proportional representation across the 11 PSROC samples is shown in Supplementary Materials, Figure S2. Ratio-of-observed-to-expected (Ro/e) analysis comparing fibroblast subpopulation distributions across surgical outcome groups demonstrated selective enrichment of the CAF-C7 subcluster (marked by high *CD34*, *C7*, and *FGF7* expression) and the MC-UPK3B subcluster (characterized by elevated *UPK3B* and *MSLN* expression) in the non-R0 group (Figure 1H). Collectively, these findings suggested that these fibroblast subsets may contribute to TME remodeling in a manner that hinders complete resection.

### 3.2. Characteristic Gene Expression and Spatial Distribution of the CAF-C7 Subpopulation in PSROC

**Figure 2:**
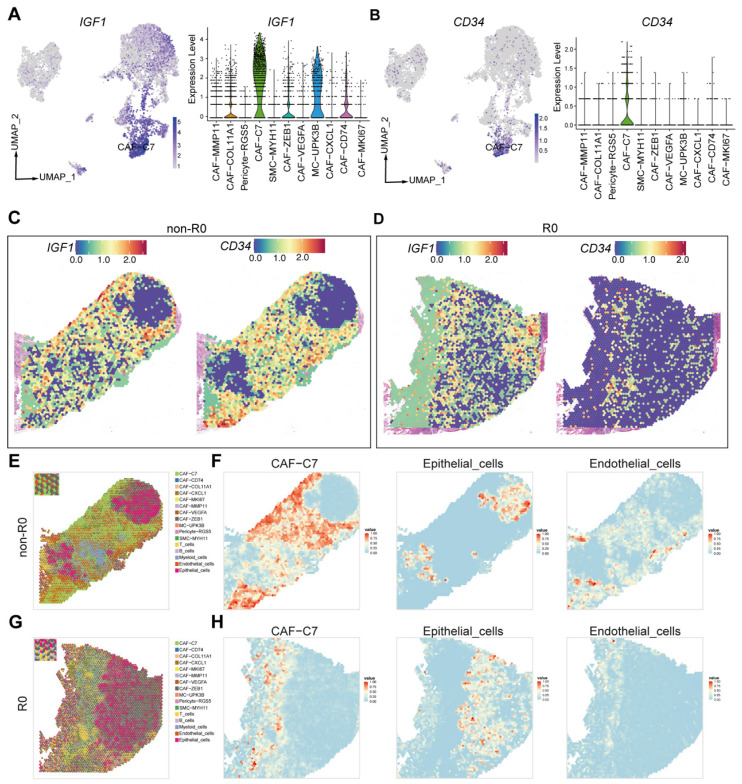
Characteristic gene expression and spatial distribution of the CAF-C7 subpopulation in PSROC. (**A**,**B**) UMAP plots and violin plots showing expression of *IGF1* (**A**) and *CD34* (**B**) across stromal subpopulations, highlighting CAF-C7 as the predominant source of *IGF1* and expressing high levels of *CD34*. (**C**,**D**) Spatial transcriptomic maps of a representative non-R0 (**C**) and R0 (**D**) tumor, visualizing expression patterns of *IGF1* and *CD34*, demonstrating enrichment of *IGF1^+^/CD34^+^* cells in non-R0 lesions. (**E**) Spatial projection of fibroblast subclusters and other cell types from non-R0 sample onto tissue sections using the CARD algorithm. (**F**) Spatial distribution of CAF-C7, epithelial cells, and endothelial cells within non-R0 sample. (**G**) Spatial projection of fibroblast subclusters and other cell types from R0 sample. (**H**) Spatial distribution of CAF-C7, epithelial cells, and endothelial cells in R0 sample.

To identify key molecular determinants associated with surgical outcomes, we performed differential gene expression analysis comparing non-R0 and R0 tumors. *IGF1* emerged as one of the most significantly upregulated genes in non-R0 tumors (Supplementary Materials, Figure S3). UMAP-based cell-type mapping further revealed that *IGF1*, a secreted factor with pleiotropic effects in the tumor microenvironment, was predominantly expressed in the CAF-C7 subpopulation (Figure 2A), suggesting a central role for CAF-C7 in non-R0 tumors. Although several genes were differentially expressed between groups, *IGF1* was prioritized because of its strong differential expression, its specific cellular origin within CAF-C7, and its well-established involvement in promoting epithelial–mesenchymal transition, angiogenesis, and therapy resistance across solid tumors [36,37,38,39].

In parallel, *CD34* enrichment was also observed in CAF-C7 and validated in Figure 1G and Figure 2B, supporting its use as a surface marker for identifying this subpopulation. To further confirm the localization and identity of CAF-C7, spatial transcriptomics was performed on matched tumor samples from one R0 and one non-R0 patient. Consistent with the single-cell findings, stromal cells in the non-R0 sample exhibited significantly higher expression of *IGF1* and *CD34* compared with those in the R0 sample (Figure 2C,D).

Using the CARD deconvolution algorithm [40], CAF subtypes and other cell types defined by scRNA-seq were projected onto two spatial transcriptomic sections (one R0 and one non-R0 sample), enabling high-resolution evaluation of their interactions with surrounding TME components. Despite the limited number of spatial samples, the observed patterns were fully consistent with those identified in the scRNA-seq cohort. Spatial transcriptomic analysis revealed distinct distribution patterns of CAF subsets across clinical outcome groups. Quantitative analysis further confirmed significant enrichment of CAF-C7 in non-R0 tumors, where it accounted for a much larger proportion of the stromal compartment compared with R0 tumors. Endothelial cells were also enriched in non-R0 tumors, indicative of enhanced angiogenic signaling within the stroma. In contrast, increased myeloid cell infiltration was observed within the tumor nests of R0 tumors, whereas the overall epithelial cell content remained largely comparable between the two groups (Figure 2E–H, Supplementary Materials, Figure S4).

### 3.3. CAF-C7 Exhibit Strengthened Interactions with Epithelial and Endothelial Cells

**Figure 3:**
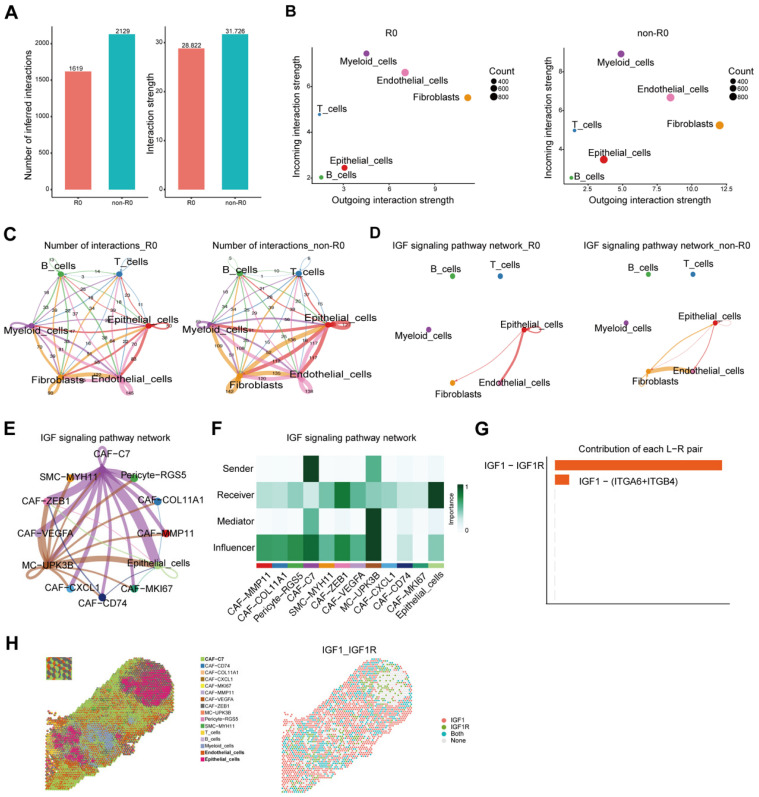
Cell–cell communication analysis. (**A**) Bar plots showing the number and strength of inferred cell–cell interactions across all cell types in R0 and non-R0 groups. Both metrics are markedly elevated in the non-R0 group. (**B**) Interaction strength of each cell type acting as either sender or receiver. Fibroblasts emerge as the dominant signal senders in both groups, while myeloid cells are the primary receivers. (**C**) Comparison of interaction counts and strengths between major cell types, highlighting stronger and more frequent interactions between fibroblasts and epithelial cells in the non-R0 group. (**D**) Group-specific representation of the IGF signaling pathway. In the non-R0 group, IGF signaling predominantly exists between fibroblasts and endothelial or epithelial cells. (**E**–**G**). IGF pathway interactions and receptor distributions between fibroblast subpopulations and epithelial cells. (**H**) Spatial transcriptomics reveal that IGF signaling interactions predominantly occur between CAF-C7 and epithelial cells in non-R0 tumors.

To characterize intercellular communication in PSROC, we conducted a comprehensive comparison of cell–cell interaction networks between R0 and non-R0 tumors using CellChat [34]. Quantitative analyses revealed a higher number and strength of intercellular interactions in the non-R0 tumor (Figure 3A), indicating a more dynamic and complex communication landscape.

Furthermore, functional signaling role analyses demonstrated that fibroblasts consistently acted as dominant signal senders, while myeloid cells served predominantly as receivers (Figure 3B). Notably, fibroblast-epithelial interactions were significantly elevated in non-R0 tumors (Figure 3C). Given that *IGF1* was significantly upregulated in CAF-C7, we further examined IGF1-mediated signaling. Our data showed that IGF pathway activation was primarily mediated via fibroblast-epithelial and fibroblast-endothelial interactions in non-R0 tumors and was largely absent in R0 tumors (Figure 3D). Ligand–receptor analysis of the IGF1-IGF1R axis demonstrated robust signaling activity between CAF-C7 and multiple fibroblast subsets. In addition, global communication network analysis identified CAF-C7 as the predominant *IGF1* ligand source and epithelial cells as primary receptors, suggesting a unidirectional and hierarchical signaling cascade (Figure 3E–G). Spatial transcriptomic profiling further demonstrated that IGF signaling interactions are predominantly localized between CAF-C7 and epithelial cells in non-R0 tumors (Figure 3H).

Taken together, these findings establish CAF-C7 as a central regulator of IGF1-mediated signaling within the PSROC TME, exerting pro-tumorigenic effects through its interactions with epithelial and endothelial cells and potentially contributing to disease progression and the difficulty in achieving complete cytoreduction(R0).

### 3.4. IGF-I Inhibits Anoikis and Promote Tumor Cell Migration in Ovarian Cancer

Anoikis is a distinct form of programmed cell death initiated upon cellular detachment from the native ECM or loss of substrate adhesion [41,42]. This mechanism is essential for maintaining tissue homeostasis and preventing aberrant cell dissemination. Resistance to anoikis constitutes a critical step in enabling tumor cells to acquire metastatic capabilities [43,44]. Thus, we hypothesized that elevated *IGF1* levels within the ovarian cancer tumor microenvironment attenuate apoptosis of detached cancer cells, thereby promoting peritoneal metastasis.

**Figure 4:**
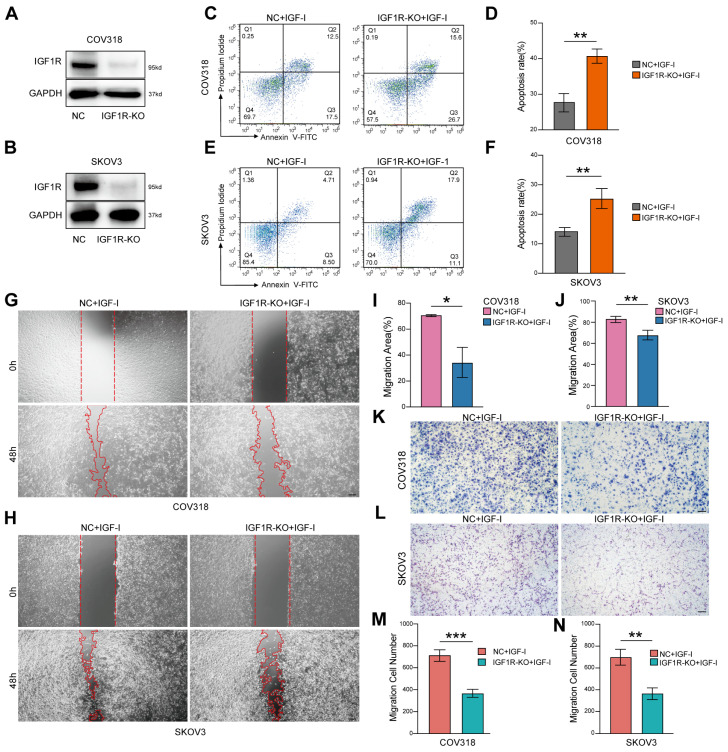
IGF-I inhibits anoikis and promotes migration in ovarian cancer cells. (**A**,**B**) Western blot analysis showing the knockout efficiency of IGF1R in COV318 (**A**) and SKOV3 (**B**) cells. (**C**) Following IGF-I treatment, anoikis levels in IGF1R knockout (KO) COV318 cells were significantly increased compared to the negative control (NC) group. (**D**) Quantitative analysis of anoikis in COV318 cells. (**E**) IGF1R knockout SKOV3 cells exhibited significantly elevated anoikis levels after IGF-I treatment compared with the NC group. (**F**) Quantitative analysis of anoikis in SKOV3 cells. (**G**,**H**) Scratch assay demonstrating that IGF-I treatment failed to promote migration in IGF1R knockout COV318 (**G**) and SKOV3 (**H**) cells. Scale bar, 200 μm. (**I**,**J**) Quantitative analysis of migration rate in COV318 (**I**) and SKOV3 (**J**) cells from scratch assay. (**K**,**L**) Trans-well migration assay further confirmed that IGF-I inhibited migration of IGF1R knockout COV318 (**K**) and SKOV3 (**L**) cells. Scale bar, 100 μm. (**M**,**N**) Quantitative analysis of Trans-well migration in COV318 (**M**) and SKOV3 (**N**) cells. Significance thresholds were defined as * *p* < 0.05, ** *p* < 0.01, and *** *p* < 0.001.

To validate the functional impact of IGF1-IGF1R signaling, we performed assays in two ovarian cancer cell lines with relatively high IGF1R expression, including the HGSOC-relevant COV318 and the widely used SKOV3 model. Using CRISPR/Cas9 gene editing, we generated IGF1R-knockout COV318 and SKOV3 cells, and successful knockout was confirmed by Western blotting (Figure 4A,B; Supplementary Materials, Figure S5). Before conducting the full set of functional assays, we first performed a baseline wound-healing experiment without the addition of IGF-I. Under this ligand-free condition, IGF1R knockout alone had almost no effect on ovarian cancer cell migration (Figure S6), suggesting that the functional consequences of IGF1R loss are largely dependent on the presence of exogenous IGF-I. Based on this observation, we conducted the subsequent experiments under IGF-I stimulation to more clearly evaluate the ligand dependence of IGF1R signaling.

Following suspension culture to simulate anoikis conditions, flow cytometric analysis demonstrated significantly increased apoptosis rates in IGF1R knockout cells compared with controls (Figure 4C–F). Subsequently, scratch wound healing and Trans-well migration assays were carried out to evaluate the role of the IGF1-IGF1R signaling axis in cell motility. IGF1R-knockout cells exhibited markedly decreased migration rates in scratch assays (Figure 4G–J), accompanied by a significant reduction in the number of migrating cells in Trans-well assays within the IGF1R-knockout cells (Figure 4K–N).

These findings demonstrate that the IGF1-IGF1R signaling pathway exerts a dual function in ovarian cancer metastasis by enhancing cell survival through inhibition of anoikis and facilitating tumor dissemination via increased cellular motility. Collectively, these results provide compelling evidence for the critical role of *IGF1* signaling in ovarian cancer progression and establish a robust experimental basis for therapeutic targeting of this pathway.

### 3.5. Induction of Tumor Angiogenesis by CAF-C7 in PSROC

**Figure 5:**
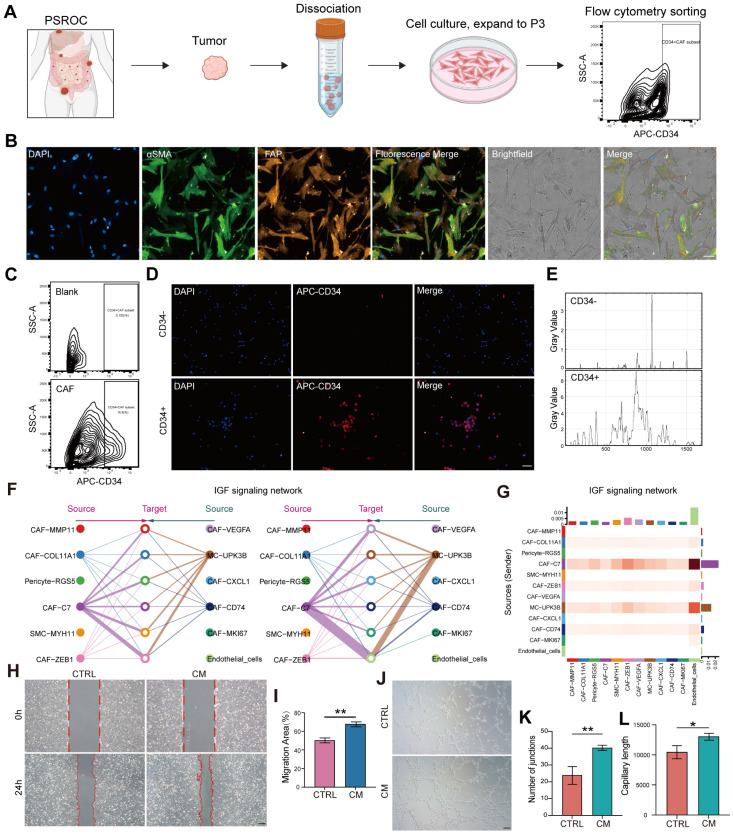
CAF-C7 conditioned medium promotes angiogenesis. (**A**) Schematic workflow of primary fibroblast isolation and flow cytometry sorting. (**B**) Immunofluorescence staining confirms the CAF phenotype, with co-expression of alpha-smooth muscle actin (α-SMA, red) and fibroblast activation protein (FAP, green); nuclei counterstained with DAPI (blue). Scale bar, 50 μm. (**C**) Flow cytometry analysis showing the proportion of CD34^+^ cells within CAF populations and the gating strategy for CD34^+^ CAF isolation. (**D**) Cytospin assay validating the sorting efficiency of CD34^+^ CAFs. Scale bar, 100 μm. (**E**) Quantification of fluorescence signals in (**D**). (**F**) Network plot visualization of interaction strength between CAF subpopulations and endothelial cells, highlighting the interaction activity of different CAF subsets with endothelial cells. (**G**) Heatmap visualization of interaction strength between CAF subpopulations and epithelial cells, showing significant IGF signaling interactions involving endothelial cells as signal receivers and the CAF-C7 and CAF-CFB subpopulations. (**H**) Conditioned medium (CM) derived from CAF-C7 significantly enhanced the migration capacity of HUVECs. Scale bar, 100 μm. (**I**) Quantitative analysis corresponding to panel (**H**). (**J**) HUVECs treated with CAF-C7 derived CM exhibited pronounced angiogenic capability, with typical ring-like structures observed in the CM-treated group. Scale bar, 100 μm. (**K**,**L**) Quantitative analysis corresponding to panel (**J**). Significance thresholds were defined as * *p* < 0.05 and ** *p* < 0.01.

To investigate the functional properties of CAF-C7, primary CAFs were isolated from ovarian cancer specimens and cultured in vitro (Figure 5A). Under bright-field microscopy, the primary cells exhibited the characteristic spindle-shaped morphology of fibroblasts (Figure 5B). Subsequently, immunofluorescence staining validated expression of canonical CAF markers, including alpha-smooth muscle actin (α-SMA) and fibroblast activation protein (FAP) (Figure 5B).

While the overexpression of CD34 in the CAF-C7 subpopulation was previously identified, significant variability in CD34 expression was observed among primary CAFs derived from different samples. This finding supported the use of CD34 as a marker for the isolation of the CAF-C7 subpopulation. Accordingly, flow cytometry was employed to isolate CD34^+^ cells from primary CAF cultures. Sorting efficiency was validated using Cytospin preparation technique assays. CD34^+^ cells exhibited distinct red fluorescence signals, while negative control samples showed no detectable fluorescence (Figure 1G and Figure 5C–E). As fibroblasts were digested using pancreatin and did not adhere during Cytospin, cells appeared to be rounded rather than spindle-shaped under microscopy. Collectively, these efforts resulted in the successful establishment of a primary CAF culture system and reliable isolation of the CAF-C7 subpopulation for subsequent functional analyses.

Cell–cell interaction analysis revealed significant crosstalk between the CAF-C7 subpopulation and vascular endothelial cells, with notably active signaling through the IGF pathway (Figure 5F,G). To validate these findings, conditioned medium (CM) derived from CAF-C7 was applied to human umbilical vein endothelial cells (HUVECs). Scratch assays demonstrated a significant enhancement in the migratory capacity of CM-treated HUVECs (Figure 5H,I). Furthermore, in vitro tube formation assays showed that HUVECs exposed to CAF-C7 CM developed denser and more structurally intact tubular networks (Figure 5J–L). These results indicate that CAF-C7-derived CM exerts potent pro-angiogenic effects, suggesting that CAF-C7 remodels the tumor vascular microenvironment via paracrine signaling.

Mechanistically, these results suggest that CAF-C7 secretes paracrine pro-angiogenic factors, including IGF-I, thereby promoting neovascularization, which supports tumor growth and invasion. These findings support the rationale for targeting the CAF-C7-IGF1 axis as a potential strategy to modulate tumor vasculature in PSROC.

## 4. Discussion

By integrating multi-omics datasets, this study elucidates the role and mechanistic basis of the CAF-C7 subpopulation in non-R0 patients undergoing secondary cytoreductive surgery (SCR) for platinum-sensitive relapsed ovarian cancer (PSROC). CAF-C7 engages in complex crosstalk with the tumor epithelial and endothelial interface through the IGF1-IGF1R signaling axis, thereby promoting anoikis resistance, enhancing metastatic potential, and facilitating angiogenesis. Collectively, these effects contribute to a multifocal recurrence phenotype and poor surgical outcomes. Our findings suggest that stromal composition, particularly the presence of CAF-C7, may be an underrecognized determinant of surgical success. This adds a molecular dimension to established clinical predictors such as the AGO criteria and the iMODEL scoring system, potentially refining patient selection for SCR and informing the development of novel stroma-targeted therapeutic strategies.

Recent landmark clinical trials, including DESKTOP-III [8] and SOC-1 [10,11], highlighted the survival benefit associated with achieving complete resection (R0) in PSROC. However, according to the current definition of optimal cytoreduction, which specifies residual tumor of 1 cm or less, patients with multifocal residual disease exhibit significantly poorer survival outcomes compared with those with unifocal residual disease. Similar trends have been reported in studies of interval debulking surgery (IDS) [45]. Considered alongside the negative findings of GOG-213 [9], these results highlight the urgent need for more refined stratification of surgical candidates, individualized surgical planning, and the timely incorporation of targeted and anti-angiogenic therapies into treatment algorithms. Clinically, achieving R0 resection is frequently limited by extensive tumor dissemination, challenging anatomical locations [46] or involvement of major vascular structures [47], all of which impose considerable technical constraints on complete surgical excision. Moreover, residual tumor cells previously exposed to multiple lines of therapy may develop enhanced proliferative and chemo-resistant phenotypes [48,49], further diminishing the effectiveness of subsequent surgical interventions. Evidence from a retrospective study of colorectal cancer liver metastases demonstrated that converting initially unresectable tumors to resectable status through conversion therapy can yield substantial survival benefits [50].

Mechanistically, CAF-C7 exhibits an inflammatory CAF (iCAF)-like phenotype, characterized by high secretory activity and elevated expression of *IGF1*, *C3*, *CD34*, *IL-6*, and *CXCL12* [23,51,52,53,54]. Distinct from myofibroblastic CAFs (myCAFs), these iCAFs are driven by inflammatory signaling and mediate the recruitment of immune cells such as macrophages and neutrophils, promote angiogenesis, and induce epithelial-to-mesenchymal transition (EMT) in cancer cells, ultimately facilitating tumor growth, metastasis, and therapeutic resistance [18,55,56,57,58]. Although *IGF1* signaling and iCAF phenotypes have been linked to therapy resistance in other tumor types, our study did not directly evaluate drug response. Future studies incorporating viability or cytotoxicity assays will be required to determine whether CAF-C7 contributes to treatment resistance in PSROC. The IGF1-IGF1R signaling pathway has been demonstrated to drive tumor progression via mechanisms including EMT induction, apoptosis inhibition, and ferroptosis resistance [59,60,61,62,63,64,65,66]. In epithelial ovarian cancer (EOC), tumor-associated macrophages promote angiogenesis via IGF-I secretion in response to angiopoietin-2 (Ang2), highlighting *IGF1* as a potential therapeutic target [67]. Additionally, hypoxia-induced senescent CAFs (hsCAFs) secrete IGF-I to suppress AMP-activated protein kinase (AMPK) activity, thereby sustaining cancer stemness and contributing to chemoresistance and adverse clinical outcomes [36]. In this study, we observed that CAF-C7, which is enriched in non-R0 tumors, expresses high levels of *IGF1*. In our in vitro model using established ovarian cancer cell lines, CAF-C7-derived IGF-I was associated with enhanced IGF1R signaling activity, improved cell survival under anoikis-inducing conditions, and increased migratory behavior. These findings suggest a potential role for CAF-C7 in modulating tumor cell responses through IGF-I; however, we acknowledge that these effects may differ in primary epithelial tumor cells owing to the intrinsic heterogeneity of ovarian cancers. Future in vivo studies will be necessary to validate the functional significance of CAF-C7-derived IGF-I within the native tumor microenvironment.

High-grade serous ovarian cancer (HGSOC) frequently spreads through the peritoneal cavity [68,69], where detached tumor cells aggregate into multicellular spheroids that resist anoikis and facilitate metastatic implantation. Soluble factors within malignant ascites are known to promote spheroid viability [70,71,72]. Our findings that IGF-I enhances anoikis resistance and cell motility suggest that IGF-I may contribute to a supportive microenvironment that enables spheroid survival and trans-coelomic dissemination. Although additional in vivo studies are needed, these data provide a mechanistic link between IGF1-IGF1R signaling and key steps of peritoneal metastatic spread in HGSOC.

The primary aim of this study was to elucidate the impact of stromal heterogeneity on surgical outcomes. By identifying CAF-C7 as a key factor associated with non-R0 resection in PSROC, we provide mechanistic insights into why some patients fail to achieve R0 resection despite favorable preoperative clinical parameters. Nevertheless, CAF-C7 is unlikely to be the sole stromal mediator of surgical resistance. Other CAF subtypes, including myofibroblastic CAFs such as CAF-MMP11 and CAF-COL11A1, as well as antigen-presenting CAFs such as CAF-CD74, may act cooperatively to shield tumor nests, increase tissue stiffness, and suppress local immune responses. In addition, angiogenic CAF-VEGFA promotes vascular expansion, and proliferative CAF-MKi67 contributes to sustained stromal turnover. A systems-level characterization of CAF subtypes and their interactions is therefore essential to fully understand tumor microenvironment states. Mechanistically, CAFs influence surgical outcomes through multiple processes. ECM remodeling induces stromal stiffening and generates challenging dissection planes [73], Pro-angiogenic signaling produces highly vascularized tumor beds, increasing intraoperative bleeding risk and complicating en bloc resection [74]. Secretion of survival factors such as IGF-I maintains the viability of residual tumor cells and enables early regrowth following incomplete resection. Together, these biological processes provide a plausible explanation for why patients with multifocal residual disease, despite meeting conventional criteria for “optimal” cytoreduction, experience significantly poorer prognoses than those with unifocal residual disease [75].

Simultaneously, IGF1-mediated angiogenesis establishes a highly vascularized niche that promotes tumor progression. These molecular alterations collectively drive a multifocal growth pattern, substantially reducing the likelihood of achieving complete cytoreduction. Notably, genetic silencing of IGF1R markedly attenuates the tumor-promoting effects of CAF-C7, underscoring IGF1R as a potential therapeutic target [37,38]. These findings have important translational implications. Diagnostically, the presence of CAF-C7 may serve as a novel biomarker to predict surgical outcomes, facilitating more precise selection of candidates for SCR. From a therapeutic perspective, IGF1R inhibitors such as linsitinib [39] may improve complete resection rates when used in a neoadjuvant setting. Furthermore, for patients with extensive multifocal recurrence who are not eligible for surgery, targeting the CAF-C7-IGF1 axis represents a promising strategy to delay disease progression.

Nonetheless, several critical questions remain unresolved. Although this study has elucidated key functional characteristics of CAF-C7, its cellular origins and lineage evolution remain incompletely understood. In addition, the interplay between CAF-C7 and other stromal or immune components within the TME requires further investigation. We note that CAF-C7 shares iCAF-like transcriptional and functional features with fibroblast subsets reported in other solid tumors, including pancreatic cancer [51], supporting the broader biological relevance of this state. At the same time, our use of prospective clinical trial samples and integrated single-cell and spatial analyses in recurrent disease provides a level of clinical and biological resolution that has been underrepresented in prior CAF studies. Future studies should focus on delineating the ontogeny of CAF-C7 using scRNA-seq and spatial transcriptomic strategies, establishing humanized models that better recapitulate treatment responses, and designing prospective studies to evaluate whether targeting IGF1-IGF1R signaling may improve surgical outcomes.

In conclusion, this study enhances our understanding of the contribution of stromal heterogeneity to surgical outcomes in relapsed ovarian cancer, highlighting CAF-C7 as a clinically relevant iCAF subset that fosters multifocal recurrence and hinders complete cytoreduction through IGF1-IGF1R signaling. By linking molecular features of the TME with surgical feasibility, our findings raise the possibility that CAF-directed strategies could complement existing therapeutic approaches. Future development of agents targeting CAF-C7, as well as thoughtfully designed combination regimens with chemotherapy, targeted therapy, or immunotherapy, may offer new avenues to improve the management of selected patients with PSROC, although dedicated translational and clinical studies will be required to validate this potential.

## Data Availability

All data reported in this paper will be shared by the lead contact upon reasonable request. Lead contact, Rongyu Zang (zang.rongyu@zs-hospital.sh.cn). This paper does not report original code.

## Supplementary Materials

The following supporting information can be downloaded at: https://www.mdpi.com/article/10.3390/biomedicines13123011/s1, Table S1: Clinical information of patients in the single-cell sequencing cohort (*n* = 11). Table S2: Clinical information of patients in the spatial transcriptomics (*n* = 2). Table S3: Clinical information of patients in the primary fibroblast cell lines (*n* = 3). Figure S1: Analysis of cell subpopulations in PSROC by scRNA-seq. Figure S2: Proportional distribution of CAF subpopulations across the 11 PSROC samples. Figure S3: Volcano plot showing differentially expressed genes between CAFs in the non-R0 and R0 groups. Figure S4: Magnified spatial transcriptomics views of tumor nest regions showing increased myeloid-cell infiltration in the R0 sample compared with the non-R0 sample. Figure S5: Quantitative analysis of IGF1R knockout (KO) Western blot bands in COV318 (A) and SKOV3 (B) cells. Figure S6: Scratch assay of IGF1R-knockout COV318 (A) and SKOV3 (B) ovarian cancer cells cultured in the absence of IGF-I [76,77].

## References

[1] Webb P.M., Jordan S.J. (2024). Global epidemiology of epithelial ovarian cancer. Nat. Rev. Clin. Oncol..

[2] Torre L.A., Trabert B., DeSantis C.E., Miller K.D., Samimi G., Runowicz C.D., Gaudet M.M., Jemal A., Siegel R.L. (2018). Ovarian cancer statistics, 2018. CA Cancer J. Clin..

[3] Kordowitzki P., Lange B., Elias K.M., Haigis M.C., Mechsner S., Braicu I.E., Sehouli J. (2025). Transforming treatment paradigms: Focus on personalized medicine for high-grade serous ovarian cancer. CA Cancer J. Clin..

[4] Rose P.G. (2022). Ovarian cancer recurrence: Is the definition of platinum sensitivity modified by PARPi, bevacizumab or other intervening treatments? A clinical perspective. Cancer Drug Resist..

[5] Du Bois A., Reuss A., Pujade-Lauraine E., Harter P., Ray-Coquard I., Pfisterer J. (2009). Role of surgical outcome as prognostic factor in advanced epithelial ovarian cancer: A combined exploratory analysis of 3 prospectively randomized phase 3 multicenter trials: By the Arbeitsgemeinschaft Gynaekologische Onkologie Studiengruppe Ovarialkarzinom (AGO-OVAR) and the Groupe d’Investigateurs Nationaux Pour les Etudes des Cancers de l’Ovaire (GINECO). Cancer.

[6] Markman M., Rothman R., Hakes T., Reichman B., Hoskins W., Rubin S., Jones W., Almadrones L., Lewis J.L. (1991). Second-line platinum therapy in patients with ovarian cancer previously treated with cisplatin. J. Clin. Oncol..

[7] Pejovic T., Fitch K., Mills G. (2022). Ovarian cancer recurrence: Is the definition of platinum resistance modified by PARP inhibitors and other intervening treatments?. Cancer Drug Resist..

[8] Harter P., Sehouli J., Vergote I., Ferron G., Reuss A., Meier W., Greggi S., Mosgaard B.J., Selle F., Guyon F. (2021). Randomized Trial of Cytoreductive Surgery for Relapsed Ovarian Cancer. N. Engl. J. Med..

[9] Coleman R.L., Spirtos N.M., Enserro D., Herzog T.J., Sabbatini P., Armstrong D.K., Kim J.W., Park S.Y., Kim B.G., Nam J.H. (2019). Secondary Surgical Cytoreduction for Recurrent Ovarian Cancer. N. Engl. J. Med..

[10] Jiang R., Feng Y., Chen Y., Cheng X., Shi T., Gao W., Jia H., Jiang S., Guo Y., Huang X. (2024). Surgery versus no surgery in platinum-sensitive relapsed ovarian cancer: Final overall survival analysis of the SOC-1 randomized phase 3 trial. Nat. Med..

[11] Shi T., Zhu J., Feng Y., Tu D., Zhang Y., Zhang P., Jia H., Huang X., Cai Y., Yin S. (2021). Secondary cytoreduction followed by chemotherapy versus chemotherapy alone in platinum-sensitive relapsed ovarian cancer (SOC-1): A multicentre, open-label, randomised, phase 3 trial. Lancet Oncol..

[12] Tian W.J., Chi D.S., Sehouli J., Tropé C.G., Jiang R., Ayhan A., Cormio G., Xing Y., Breitbach G.P., Braicu E.I. (2012). A risk model for secondary cytoreductive surgery in recurrent ovarian cancer: An evidence-based proposal for patient selection. Ann. Surg. Oncol..

[13] Sahai E., Astsaturov I., Cukierman E., DeNardo D.G., Egeblad M., Evans R.M., Fearon D., Greten F.R., Hingorani S.R., Hunter T. (2020). A framework for advancing our understanding of cancer-associated fibroblasts. Nat. Rev. Cancer.

[14] Biffi G., Tuveson D.A. (2021). Diversity and Biology of Cancer-Associated Fibroblasts. Physiol. Rev..

[15] Lavie D., Ben-Shmuel A., Erez N., Scherz-Shouval R. (2022). Cancer-associated fibroblasts in the single-cell era. Nat. Cancer.

[16] Chen Y., McAndrews K.M., Kalluri R. (2021). Clinical and therapeutic relevance of cancer-associated fibroblasts. Nat. Rev. Clin. Oncol..

[17] Caligiuri G., Tuveson D.A. (2023). Activated fibroblasts in cancer: Perspectives and challenges. Cancer Cell.

[18] Kennel K.B., Bozlar M., De Valk A.F., Greten F.R. (2023). Cancer-Associated Fibroblasts in Inflammation and Antitumor Immunity. Clin. Cancer Res..

[19] Alexandre Y.O., Schienstock D., Lee H.J., Gandolfo L.C., Williams C.G., Devi S., Pal B., Groom J.R., Cao W., Christo S.N. (2022). A diverse fibroblastic stromal cell landscape in the spleen directs tissue homeostasis and immunity. Sci. Immunol..

[20] Buechler M.B., Pradhan R.N., Krishnamurty A.T., Cox C., Calviello A.K., Wang A.W., Yang Y.A., Tam L., Caothien R., Roose-Girma M. (2021). Cross-tissue organization of the fibroblast lineage. Nature.

[21] Shi Y., Gao W., Lytle N.K., Huang P., Yuan X., Dann A.M., Ridinger-Saison M., DelGiorno K.E., Antal C.E., Liang G. (2019). Targeting LIF-mediated paracrine interaction for pancreatic cancer therapy and monitoring. Nature.

[22] Micalet A., Upadhyay A., Javanmardi Y., de Brito C.G., Moeendarbary E., Cheema U. (2024). Patient-specific colorectal-cancer-associated fibroblasts modulate tumor microenvironment mechanics. iScience.

[23] Cords L., Engler S., Haberecker M., Rüschoff J.H., Moch H., de Souza N., Bodenmiller B. (2024). Cancer-associated fibroblast phenotypes are associated with patient outcome in non-small cell lung cancer. Cancer Cell.

[24] Grout J.A., Sirven P., Leader A.M., Maskey S., Hector E., Puisieux I., Steffan F., Cheng E., Tung N., Maurin M. (2022). Spatial Positioning and Matrix Programs of Cancer-Associated Fibroblasts Promote T-cell Exclusion in Human Lung Tumors. Cancer Discov..

[25] Dominguez C.X., Müller S., Keerthivasan S., Koeppen H., Hung J., Gierke S., Breart B., Foreman O., Bainbridge T.W., Castiglioni A. (2020). Single-Cell RNA Sequencing Reveals Stromal Evolution into LRRC15(+) Myofibroblasts as a Determinant of Patient Response to Cancer Immunotherapy. Cancer Discov..

[26] Elyada E., Bolisetty M., Laise P., Flynn W.F., Courtois E.T., Burkhart R.A., Teinor J.A., Belleau P., Biffi G., Lucito M.S. (2019). Cross-Species Single-Cell Analysis of Pancreatic Ductal Adenocarcinoma Reveals Antigen-Presenting Cancer-Associated Fibroblasts. Cancer Discov..

[27] Costa A., Kieffer Y., Scholer-Dahirel A., Pelon F., Bourachot B., Cardon M., Sirven P., Magagna I., Fuhrmann L., Bernard C. (2018). Fibroblast Heterogeneity and Immunosuppressive Environment in Human Breast Cancer. Cancer Cell.

[28] Kieffer Y., Hocine H.R., Gentric G., Pelon F., Bernard C., Bourachot B., Lameiras S., Albergante L., Bonneau C., Guyard A. (2020). Single-Cell Analysis Reveals Fibroblast Clusters Linked to Immunotherapy Resistance in Cancer. Cancer Discov..

[29] Gao Y., Li J., Cheng W., Diao T., Liu H., Bo Y., Liu C., Zhou W., Chen M., Zhang Y. (2024). Cross-tissue human fibroblast atlas reveals myofibroblast subtypes with distinct roles in immune modulation. Cancer Cell.

[30] Vázquez-García I., Uhlitz F., Ceglia N., Lim J.L.P., Wu M., Mohibullah N., Niyazov J., Ruiz A.E.B., Boehm K.M., Bojilova V. (2022). Ovarian cancer mutational processes drive site-specific immune evasion. Nature.

[31] Zheng X., Wang X., Cheng X., Liu Z., Yin Y., Li X., Huang Z., Wang Z., Guo W., Ginhoux F. (2023). Single-cell analyses implicate ascites in remodeling the ecosystems of primary and metastatic tumors in ovarian cancer. Nat. Cancer.

[32] Zhang L., Yu X., Zheng L., Zhang Y., Li Y., Fang Q., Gao R., Kang B., Zhang Q., Huang J.Y. (2018). Lineage tracking reveals dynamic relationships of T cells in colorectal cancer. Nature.

[33] Guo X., Zhang Y., Zheng L., Zheng C., Song J., Zhang Q., Kang B., Liu Z., Jin L., Xing R. (2018). Global characterization of T cells in non-small-cell lung cancer by single-cell sequencing. Nat. Med..

[34] Jin S., Plikus M.V., Nie Q. (2025). CellChat for systematic analysis of cell-cell communication from single-cell transcriptomics. Nat. Protoc..

[35] Becht E., Giraldo N.A., Lacroix L., Buttard B., Elarouci N., Petitprez F., Selves J., Laurent-Puig P., Sautès-Fridman C., Fridman W.H. (2016). Estimating the population abundance of tissue-infiltrating immune and stromal cell populations using gene expression. Genome Biol..

[36] Ou Z., Zhu L., Chen X., Liu T., Cheng G., Liu R., Zhang S., Tan W., Lin D., Wu C. (2025). Hypoxia-Induced Senescent Fibroblasts Secrete IGF1 to Promote Cancer Stemness in Esophageal Squamous Cell Carcinoma. Cancer Res..

[37] de Billy E., Pellegrino M., Orlando D., Pericoli G., Ferretti R., Businaro P., Ajmone-Cat M.A., Rossi S., Petrilli L.L., Maestro N. (2022). Dual IGF1R/IR inhibitors in combination with GD2-CAR T-cells display a potent anti-tumor activity in diffuse midline glioma H3K27M-mutant. Neuro Oncol..

[38] Bruchim I., Sarfstein R., Reiss A., Flescher E., Werner H. (2014). IGF1R tyrosine kinase inhibitor enhances the cytotoxic effect of methyl jasmonate in endometrial cancer. Cancer Lett..

[39] Yuan Y., Levy S.M., Yeo Y.Q., Shayan R., Karnezis T., Stacker S.A., Achen M.G. (2025). Targeting insulin-like growth factor 1 receptor restricts development and severity of secondary lymphedema in mice. iScience.

[40] Ma Y., Zhou X. (2022). Spatially informed cell-type deconvolution for spatial transcriptomics. Nat. Biotechnol..

[41] Wang Y., Cheng S., Fleishman J.S., Chen J., Tang H., Chen Z.S., Chen W., Ding M. (2024). Targeting anoikis resistance as a strategy for cancer therapy. Drug Resist. Updat..

[42] Gilmore A.P. (2005). Anoikis. Cell Death Differ..

[43] Dai Y., Zhang X., Ou Y., Zou L., Zhang D., Yang Q., Qin Y., Du X., Li W., Yuan Z. (2023). Anoikis resistance–protagonists of breast cancer cells survive and metastasize after ECM detachment. Cell Commun. Signal.

[44] Liu M., Yang J., Xu B., Zhang X. (2021). Tumor metastasis: Mechanistic insights and therapeutic interventions. MedComm.

[45] Manning-Geist B.L., Hicks-Courant K., Gockley A.A., Clark R.M., Del Carmen M.G., Growdon W.B., Horowitz N.S., Berkowitz R.S., Muto M.G., Worley M.J. (2019). A novel classification of residual disease after interval debulking surgery for advanced-stage ovarian cancer to better distinguish oncologic outcome. Am. J. Obstet. Gynecol..

[46] Stoop T.F., Theijse R.T., Seelen L.W.F., Groot Koerkamp B., van Eijck C.H.J., Wolfgang C.L., van Tienhoven G., van Santvoort H.C., Molenaar I.Q., Wilmink J.W. (2024). Preoperative chemotherapy, radiotherapy and surgical decision-making in patients with borderline resectable and locally advanced pancreatic cancer. Nat. Rev. Gastroenterol. Hepatol..

[47] Dholakia A.S., Hacker-Prietz A., Wild A.T., Raman S.P., Wood L.D., Huang P., Laheru D.A., Zheng L., De Jesus-Acosta A., Le D.T. (2013). Resection of borderline resectable pancreatic cancer after neoadjuvant chemoradiation does not depend on improved radiographic appearance of tumor-vessel relationships. J. Radiat. Oncol..

[48] Skourti E., Seip K., Mensali N., Jabeen S., Juell S., Øynebråten I., Pettersen S., Engebraaten O., Corthay A., Inderberg E.M. (2025). Chemoresistant tumor cell secretome potentiates immune suppression in triple negative breast cancer. Breast Cancer Res..

[49] Hara J., Miyata H., Yamasaki M., Sugimura K., Takahashi T., Kurokawa Y., Nakajima K., Takiguchi S., Mori M., Doki Y. (2014). Mesenchymal phenotype after chemotherapy is associated with chemoresistance and poor clinical outcome in esophageal cancer. Oncol. Rep..

[50] Basso M., Dadduzio V., Ardito F., Lombardi P., Strippoli A., Vellone M., Orlandi A., Rossi S., Cerchiaro E., Cassano A. (2016). Conversion Chemotherapy for Technically Unresectable Colorectal Liver Metastases: A Retrospective, STROBE-Compliant, Single-Center Study Comparing Chemotherapy Alone and Combination Chemotherapy with Cetuximab or Bevacizumab. Medicine.

[51] Öhlund D., Handly-Santana A., Biffi G., Elyada E., Almeida A.S., Ponz-Sarvise M., Corbo V., Oni T.E., Hearn S.A., Lee E.J. (2017). Distinct populations of inflammatory fibroblasts and myofibroblasts in pancreatic cancer. J. Exp. Med..

[52] Givel A.M., Kieffer Y., Scholer-Dahirel A., Sirven P., Cardon M., Pelon F., Magagna I., Gentric G., Costa A., Bonneau C. (2018). miR200-regulated CXCL12β promotes fibroblast heterogeneity and immunosuppression in ovarian cancers. Nat. Commun..

[53] Chen Z., Zhou L., Liu L., Hou Y., Xiong M., Yang Y., Hu J., Chen K. (2020). Single-cell RNA sequencing highlights the role of inflammatory cancer-associated fibroblasts in bladder urothelial carcinoma. Nat. Commun..

[54] Cords L., Tietscher S., Anzeneder T., Langwieder C., Rees M., de Souza N., Bodenmiller B. (2023). Cancer-associated fibroblast classification in single-cell and spatial proteomics data. Nat. Commun..

[55] Nicolas A.M., Pesic M., Engel E., Ziegler P.K., Diefenhardt M., Kennel K.B., Buettner F., Conche C., Petrocelli V., Elwakeel E. (2022). Inflammatory fibroblasts mediate resistance to neoadjuvant therapy in rectal cancer. Cancer Cell.

[56] Rizzolio S., Giordano S., Corso S. (2022). The importance of being CAFs (in cancer resistance to targeted therapies). J. Exp. Clin. Cancer Res..

[57] Faa G., Ziranu P., Pretta A., Cau F., Castagnola M., Spanu D., Saba G., D’Agata A.P., Tiwari E., Suri J.S. (2025). Cancer-associated fibroblasts (CAFs) and plaque-associated fibroblasts (PAFs): Unraveling the cellular crossroads of atherosclerosis and cancer. Biomed. Pharmacother..

[58] Parte S., Kaur A.B., Nimmakayala R.K., Ogunleye A.O., Chirravuri R., Vengoji R., Leon F., Nallasamy P., Rauth S., Alsafwani Z.W. (2024). Cancer-Associated Fibroblast Induces Acinar-to-Ductal Cell Transdifferentiation and Pancreatic Cancer Initiation Via LAMA5/ITGA4 Axis. Gastroenterology.

[59] Gui R., Li W., Li Z., Wang H., Wu Y., Jiao W., Zhao G., Shen Y., Wang L., Zhang J. (2023). Effects and potential mechanisms of IGF1/IGF1R in the liver fibrosis: A review. Int. J. Biol. Macromol..

[60] Hakuno F., Takahashi S.I. (2018). IGF1 receptor signaling pathways. J. Mol. Endocrinol..

[61] Bayne M.L., Applebaum J., Underwood D., Chicchi G.G., Green B.G., Hayes N.S., Cascieri M.A. (1989). The C region of human insulin-like growth factor (IGF) I is required for high affinity binding to the type 1 IGF receptor. J. Biol. Chem..

[62] Matsushita M., Fujita K., Hayashi T., Kayama H., Motooka D., Hase H., Jingushi K., Yamamichi G., Yumiba S., Tomiyama E. (2021). Gut Microbiota-Derived Short-Chain Fatty Acids Promote Prostate Cancer Growth via IGF1 Signaling. Cancer Res..

[63] Sorrentino C., D’Antonio L., Ciummo S.L., Fieni C., Landuzzi L., Ruzzi F., Vespa S., Lanuti P., Lotti L.V., Lollini P.L. (2022). CRISPR/Cas9-mediated deletion of Interleukin-30 suppresses IGF1 and CXCL5 and boosts SOCS3 reducing prostate cancer growth and mortality. J. Hematol. Oncol..

[64] Liu H., Gao J., Feng M., Cheng J., Tang Y., Cao Q., Zhao Z., Meng Z., Zhang J., Zhang G. (2024). Integrative molecular and spatial analysis reveals evolutionary dynamics and tumor-immune interplay of in situ and invasive acral melanoma. Cancer Cell.

[65] Fang K., Sun M., Leng Z., Chu Y., Zhao Z., Li Z., Zhang Y., Xu A., Zhang Z., Zhang L. (2023). Targeting IGF1R signaling enhances the sensitivity of cisplatin by inhibiting proline and arginine metabolism in oesophageal squamous cell carcinoma under hypoxia. J. Exp. Clin. Cancer Res..

[66] Wu K., Yan M., Liu T., Wang Z., Duan Y., Xia Y., Ji G., Shen Y., Wang L., Li L. (2023). Creatine kinase B suppresses ferroptosis by phosphorylating GPX4 through a moonlighting function. Nat. Cell Biol..

[67] Wang X., Zhu Q., Lin Y., Wu L., Wu X., Wang K., He Q., Xu C., Wan X., Wang X. (2017). Crosstalk between TEMs and endothelial cells modulates angiogenesis and metastasis via IGF1-IGF1R signalling in epithelial ovarian cancer. Br. J. Cancer.

[68] Quiralte M., Barquín A., Yagüe-Fernández M., Navarro P., Grazioso T.P., Sevillano-Fernández E., Rodriguez-Moreno J.F., Balarezo-Saldivar A., Peinado H., Izquierdo E. (2024). Proteomic profiles of peritoneal fluid-derived small extracellular vesicles correlate with patient outcome in ovarian cancer. J. Clin. Investig..

[69] Fogg K.C., Olson W.R., Miller J.N., Khan A., Renner C., Hale I., Weisman P.S., Kreeger P.K. (2019). Alternatively activated macrophage-derived secretome stimulates ovarian cancer spheroid spreading through a JAK2/STAT3 pathway. Cancer Lett..

[70] Heidari S., Kolahdouz-Mohammadi R., Khodaverdi S., Tajik N., Delbandi A.A. (2021). Expression levels of MCP-1, HGF, and IGF-1 in endometriotic patients compared with non-endometriotic controls. BMC Womens Health.

[71] Giudice L.C., Dsupin B.A., Gargosky S.E., Rosenfeld R.G., Irwin J.C. (1994). The insulin-like growth factor system in human peritoneal fluid: Its effects on endometrial stromal cells and its potential relevance to endometriosis. J. Clin. Endocrinol. Metab..

[72] Koutsilieris M., Akoum A., Lazure C., Frenette G., Lemay A. (1995). N-terminal truncated forms of insulin-like growth factor binding protein-3 in the peritoneal fluid of women without laparoscopic evidence of endometriosis. Le groupe d’investigation en gynécologie. Fertil. Steril..

[73] Wang K., Ning S., Zhang S., Jiang M., Huang Y., Pei H., Li M., Tan F. (2025). Extracellular matrix stiffness regulates colorectal cancer progression via HSF4. J. Exp. Clin. Cancer Res..

[74] Baghaie L., Haxho F., Leroy F., Lewis B., Wawer A., Minhas S., Harless W.W., Szewczuk M.R. (2023). Contemporaneous Perioperative Inflammatory and Angiogenic Cytokine Profiles of Surgical Breast, Colorectal, and Prostate Cancer Patients: Clinical Implications. Cells.

[75] Quesada S., Thomas Q.D., Colombo P.E., Fiteni F. (2023). Optimal First-Line Medico-Surgical Strategy in Ovarian Cancers: Are We There Yet?. Cancers.

[76] Hao Y., Hao S., Andersen-Nissen E., Mauck W.M., Zheng S., Butler A., Lee M.J., Wilk A.J., Darby C., Zager M. (2021). Integrated analysis of multimodal single-cell data. Cell.

[77] Jin S., Guerrero-Juarez C.F., Zhang L., Chang I., Ramos R., Kuan C.H., Myung P., Plikus M.V., Nie Q. (2021). Inference and analysis of cell-cell communication using CellChat. Nat. Commun..

