# Supplementary material for: Single-Cell Analysis Links C7+ Cancer-Associated Fibroblasts with Incomplete Resection in Platinum-Sensitive Relapsed Ovarian Cancer
Source: Biomedicines. 2025 Dec 8;13(12):3011. doi: 10.3390/biomedicines13123011 (PMC12731083; doi:10.3390/biomedicines13123011)
Supplement: Supplementary file 1 [file biomedicines-13-03011-s001.zip › Supplementary Figures.pdf]

Single-cell analysis links C7+ cancer-associated fibroblasts with incomplete resection in platinum-sensitive relapsed ovarian cancer

Longxia Li [1, 2†], Shangbing Gao [1†], Yilizhati Maimaiti [1†], Lifeng Lin [1], Xiaoxia Xing [1], Wei Wu [1], Yulian Chen [1], Mei-Chun Cai [2*], Guanglei Zhuang [2*] and Rongyu Zang [1*]

[1] Institute for Ovarian Cancer, Fudan University & Department of Gynecologic Oncology, Cancer Center, Fudan University Zhongshan Hospital, Shanghai 200032, China; llx_sync@163.com (L.L.).
[2] Shanghai Key Laboratory of Gynecologic Oncology, Ren Ji Hospital, Shanghai Jiao Tong University School of Medicine, Shanghai 200127, China.
* Correspondence: caimeichun@renji.com (M.C.); zhuangguanglei@gmail.com (G.Z.); zang.rongyu@zs-hospital.sh.cn (R.Z.).
† These authors contributed equally to this work.

Supplementary Materials

The following supporting information can be downloaded at: https://www.mdpi.com/
Table S1: Clinical information of patients in the single-cell sequencing cohort (n = 11)
Table S2: Clinical information of patients in the spatial transcriptomics (n = 2)
Table S3: Clinical information of patients in the primary fibroblast cell lines (n = 3)
Figure S1: Analysis of cell subpopulations in PSROC by scRNA-seq.
Figure S2. Proportional distribution of CAF subpopulations across the 11 PSROC samples.
Figure S3: Volcano plot showing differentially expressed genes between CAFs in the non-R0 and R0 groups.
Figure S4. Magnified spatial transcriptomics views of tumor nest regions showing increased myeloid-cell infiltration in the R0 sample compared with the non-R0 sample.
Figure S5. Quantitative analysis of IGF1R knockout (KO) Western blot bands in COV318 (A) and SKOV3 (B) cells.
Figure S6. Scratch assay of IGF1R-knockout COV318 (A) and SKOV3 (B) ovarian cancer cells cultured in the absence of IGF-I.
KEY RESOURCES TABLE

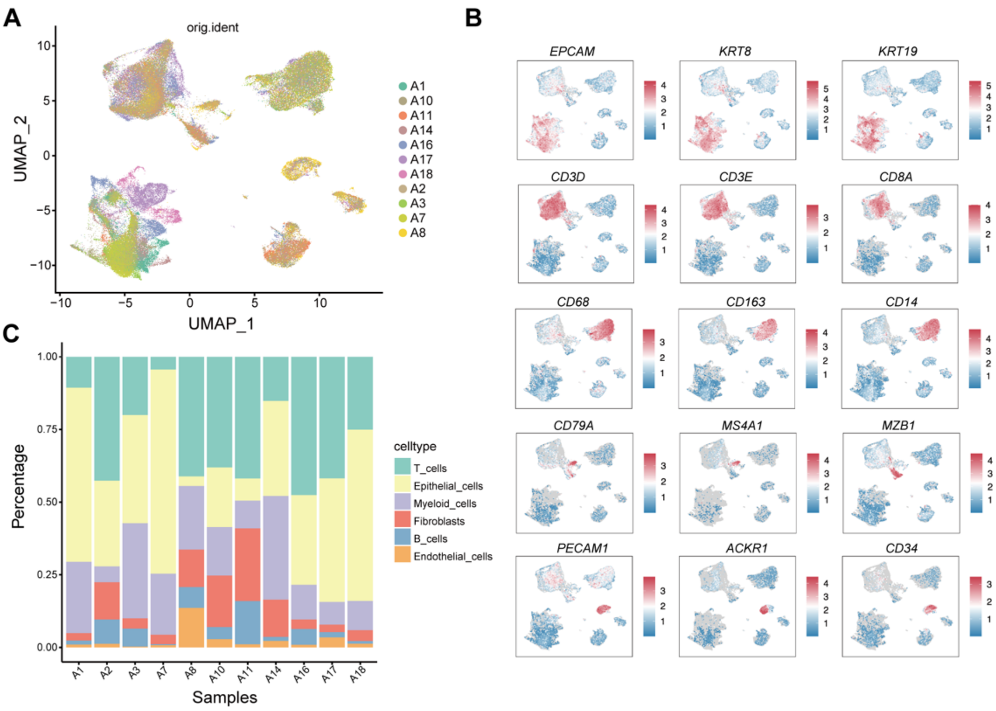

Figure S1. Analysis of cell subpopulations in PSROC by scRNA-seq. (**A**) Integrated single-cell analysis results from 11 PSROC samples. (**B**) Expression patterns of representative highly expressed genes across the five major cell clusters identified in PSROC samples. (**C**) Proportions of six major cell types in each of the 11 samples.

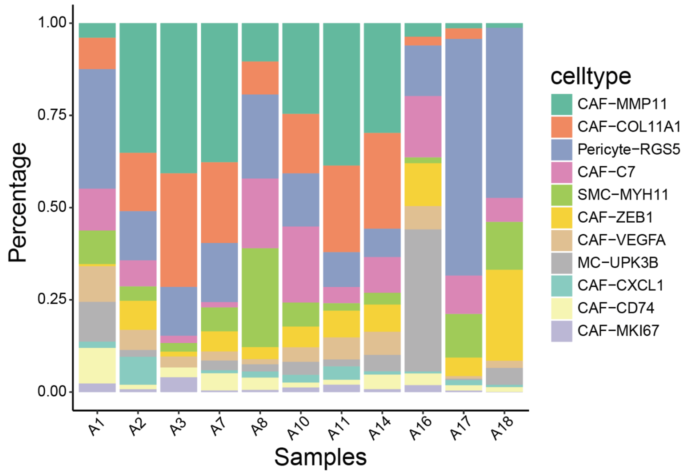

Figure S2. Proportional distribution of CAF subpopulations across the 11 PSROC samples.

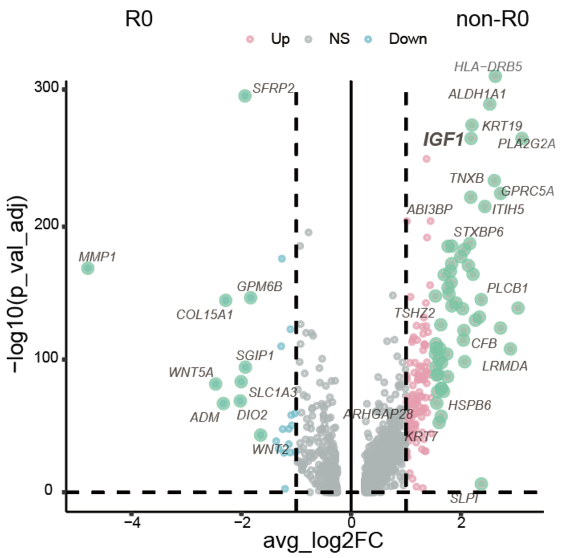

Figure S3. Volcano plot showing differentially expressed genes between CAFs in the non-R0 and R0 groups. Upregulated genes are shown in red, downregulated genes in blue, and non-significant genes in grey. Significantly dysregulated genes highlighted for emphasis are marked with green circles.

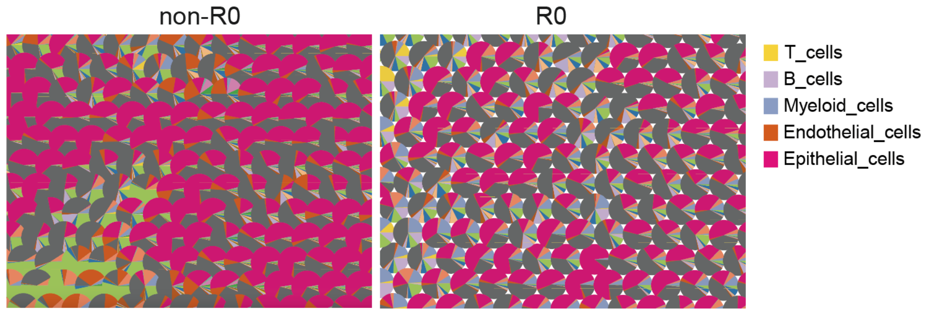

Figure S4. Magnified spatial transcriptomics views of tumor nest regions showing increased myeloid-cell infiltration in the R0 sample compared with the non-R0 sample.

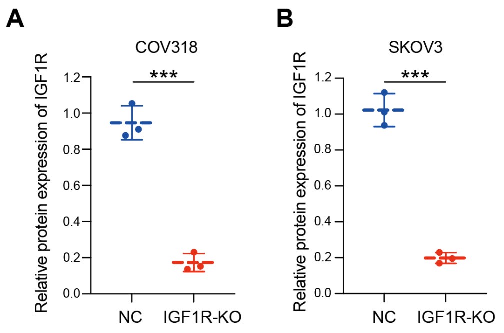

Figure S5. Quantitative analysis of IGF1R knockout (KO) Western blot bands in COV318 (A) and SKOV3 (B) cells.

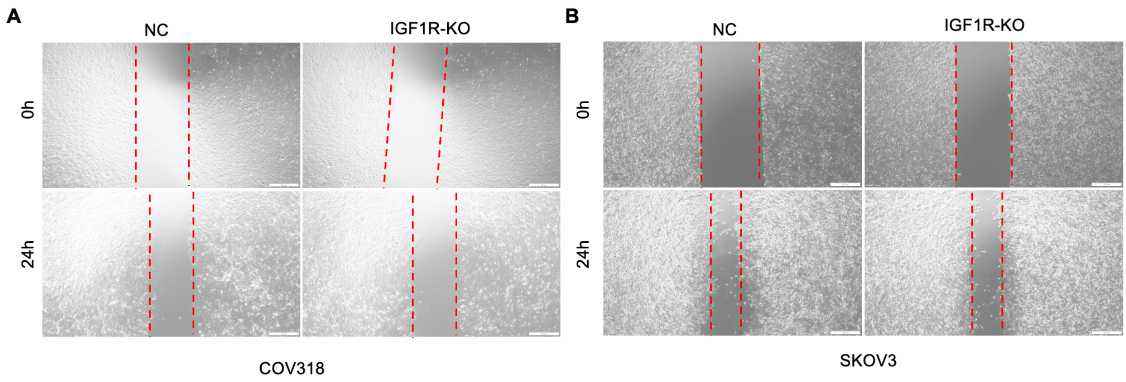

Figure S6. Scratch assay of IGF1R-knockout COV318 (**A**) and SKOV3 (**B**) ovarian cancer cells cultured in the absence of IGF-I.

KEY RESOURCES TABLE

REAGENT or RESOURCE	SOURCE	IDENTIFIER
Antibodies		
αSMA	Cell Signaling Technology	Cat#19245; RRID: AB_2734735
FAP	Abcam	#ab218164
IGF1R	Cell Signaling Technology	Cat#9750; RRID: AB_10950969
APC-conjugated anti-human CD34 antibody	BioLegend	Cat# S20016E
HRP-conjugated secondary antibodies	Beyotime	Cat#A0201
Experimental models: Cell lines		
Human: COV318	Sigma Aldrich	Cat# 07071903
Human: SKOV3	ATCC	Cat#HTB-77
HEK293T	ATCC	Cat#CRL-3216
Chemicals, peptides, and recombinant proteins		
BSA	Beyotime	Cat#ST023
IGF-I Protein, Human (70a.a,)	MedChemExpress	Cat#HY-P7018
Matrigel matrix	Corning	Cat#354237
Critical commercial assays		
Human Tumor Dissociation Kit	Miltenyi Biotec	Cat#130-095-929
ECL Chemiluminescent Substrate Kit	Yeasen Biotechnology	Cat#36222ES60
Oligonucleotides		
sgIGF1R: CTCTCGC TCTGGCCGACGAG	This paper	N/A
Software and algorithms		
Cell Ranger (v7.1.0)	10x Genomics	https://10xgenomics.com/support/cn/software/cell-ranger
Seurat (v4.1.0)	Hao et al.,[1]	https://satijalab.org/seurat
Space Ranger (v2.1.1)	10x Genomics	N/A

R (4.4.1)	N/A	https://www.r-project.org/
CellChat (R package)	https://github.com/sqjin/CellChat[2]	RRID:SCR_021946
CARD (R package)	https://github.com/YMa-lab/CARD[3]	N/A
Biorender	https://biorender.com	RRID:SCR_018361
ImageJ (Fiji)	NIH	N/A
Prism GraphPad (v. 9.0)	Prism GraphPad	https://www.graphpad.com/features
FlowJo (v. 10.8.1)	N/A	FlowJo, LLC

1. Hao, Y.; Hao, S.; Andersen-Nissen, E.; Mauck, W.M., 3rd; Zheng, S.; Butler, A.; Lee, M.J.; Wilk, A.J.; Darby, C.; Zager, M.; et al. Integrated analysis of multimodal single-cell data. *Cell* **2021**, *184*, 3573-3587.e3529, doi:10.1016/j.cell.2021.04.048.
2. Jin, S.; Guerrero-Juarez, C.F.; Zhang, L.; Chang, I.; Ramos, R.; Kuan, C.H.; Myung, P.; Plikus, M.V.; Nie, Q. Inference and analysis of cell-cell communication using CellChat. *Nat Commun* **2021**, *12*, 1088, doi:10.1038/s41467-021-21246-9.
3. Ma, Y.; Zhou, X. Spatially informed cell-type deconvolution for spatial transcriptomics. *Nat Biotechnol* **2022**, *40*, 1349-1359, doi:10.1038/s41587-022-01273-7.
